# Serum free eicosapentaenoic acid-to-arachidonic acid ratio and long-term risk of major adverse cardiovascular events in patients with coronary artery disease

**DOI:** 10.3389/fcvm.2026.1944383

**Published:** 2026-09-29

**Authors:** Changguo Ou, Wenli Wang, Dongliang Liang, Jiangxiong Wen, Zhiwen Xu, Yuli Huang, Jianqiu Liang, Jiandi Wu

**Affiliations:** 1 The First Clinical Medical College of Guangdong Medical University, Zhanjiang, China; 2 Department of Cardiology, Foshan Second People’s Hospital, Foshan, China; 3 Department of Cardiology, Eighth Affiliated Hospital of Southern Medical University, Foshan, China

**Keywords:** coronary artery disease, free EPA/AA ratio, major adverse cardiovascular events, non-esterified fatty acids, prognosis

## Abstract

**Background:**

The eicosapentaenoic acid to arachidonic acid (EPA/AA) ratio reflects the balance between n-3 and n-6 polyunsaturated fatty acids. We examined the association between the serum free (non-esterified) EPA/AA ratio and long-term major adverse cardiovascular events (MACE) in patients with angiographically confirmed coronary artery disease (CAD).

**Methods:**

This single-center retrospective cohort study included 308 patients with CAD. Serum free fatty acids were quantified by LC-MS/MS, and the free EPA/AA ratio was evaluated as the primary fatty-acid biomarker. Cox proportional hazards models were used to estimate hazard ratios (HRs), and principal component analysis (PCA) was used to explore fatty-acid patterns.

**Results:**

During a median follow-up of 48.7 months, 201 first MACE events occurred. In the fully adjusted primary analysis, each 1-SD increase in the free EPA/AA ratio was associated with a lower risk of MACE (HR = 0.82, 95% CI 0.70–0.96, *P* = 0.011; FDR-adjusted *P* = 0.049). The association was attenuated after additional adjustment for analytical batch (HR = 0.92, 95% CI 0.77–1.10, *P* = 0.375) and in a Cox model stratified by batch (HR = 0.92, 95% CI 0.77–1.11, *P* = 0.398).

**Conclusions:**

A higher serum free EPA/AA ratio was associated with a lower risk of MACE in the primary multivariable analysis, but the association was attenuated in additional sensitivity analyses. These findings should be interpreted cautiously and require validation in independent cohorts.

## Introduction

Coronary artery disease (CAD) remains a major contributor to cardiovascular morbidity and mortality worldwide and in China (1–3). Although guideline-directed medical therapy, revascularization, and secondary prevention have improved outcomes, patients with established CAD remain at risk for recurrent cardiovascular events (4, 5). Circulating biomarkers that reflect residual metabolic and inflammatory risk may therefore help refine long-term risk stratification.

Fatty acids are important components of energy metabolism and cell membranes and are involved in inflammatory activation, endothelial dysfunction, thrombosis, and atherosclerotic progression (6, 7). Previous studies have linked dietary and circulating fatty acids to coronary heart disease risk (8–16). However, evidence regarding long-term prognosis in patients with angiographically confirmed CAD remains limited.

The EPA/AA ratio represents the balance between the n-3 fatty acid EPA and the n-6 fatty acid AA. Observational studies have related EPA/AA measures or other omega-3 biomarkers to coronary outcomes (17–19), and the clinical utility of the EPA/AA ratio has also been reviewed (20). Intervention trials of EPA or omega-3 formulations have yielded heterogeneous results (21–23). However, measurement approaches differ across studies, and many established EPA/AA data are based on total serum or plasma fatty acids rather than the free (non-esterified) fatty-acid fraction. The prognostic relevance of a serum free EPA/AA ratio in established CAD is less well defined.

In this study, we examined the association between the serum free EPA/AA ratio and long-term MACE in patients with angiographically confirmed CAD. We also assessed PCA-derived fatty-acid patterns and performed spline, subgroup, and sensitivity analyses to evaluate the consistency of the association.

## Methods

### Study design and participants

This was a single-center retrospective cohort study with follow-up. The study database comprised patients who had provided written informed consent and had an adequate stored fasting serum sample available for fatty-acid measurement; it was not intended to represent a consecutive series of all patients undergoing coronary angiography during the study period. A total of 311 patients were initially screened from the study database. After excluding three patients without angiographically confirmed CAD, 308 patients with CAD were included. The index hospitalization dates ranged from April 2, 2020 to October 10, 2023. Baseline demographics, cardiovascular risk factors, laboratory measurements, echocardiographic parameters, coronary lesion severity, and follow-up outcomes were extracted from the database.

### Ethics

The study involving human participants was reviewed and approved by the Ethics Committee of Foshan Second People's Hospital (No. (2022)-0066). All participants provided written informed consent. The study was conducted in accordance with the Declaration of Helsinki.

### Fatty acid measurement and exposure definitions

Venous blood samples were collected after fasting for at least 8 h and before coronary angiography and procedural heparin administration. Serum was stored at −80 °C until analysis. Serum free (non-esterified) fatty acids were quantified using liquid chromatography-tandem mass spectrometry (LC-MS/MS). No hydrolysis step was performed.

For sample preparation, 50 μL of thawed serum was extracted with 150 μL isopropanol/acetonitrile (1:1) for 30 min and centrifuged at 12,000 rpm for 10 min at 4 °C. A 95-μL aliquot of supernatant was mixed with 5 μL of internal-standard solution containing FFA19:0 and FFA16:0-D4 (25 μg/mL each). Chromatographic separation was performed using a Waters ACQUITY UPLC I-Class system with a Waters UPLC BEH C8 column (2.1 × 100 mm, 1.7 μm) at 55 °C. Mobile phase A was acetonitrile/water (1:10) containing 0.1% acetic acid and 1 mM ammonium acetate, and mobile phase B was isopropanol/acetonitrile (1:1). The flow rate was 0.26 mL/min and the injection volume was 5 μL. The gradient for phase B was 10% at 0 min, 35% at 2 min, 85% at 4 min, 100% at 6–7.5 min, and 10% at 7.6–9 min. Detection was performed with a Waters Xevo TQ-S Micro tandem quadrupole mass spectrometer in negative electrospray ionization mode. Targeted peak areas were processed using TargetLynx, and concentrations were obtained by single-point internal-standard quantification. The same analytical protocol was used for both analytical batches.

The free EPA/AA ratio was calculated as the measured serum free EPA C20:5 concentration divided by the serum free AA C20:4 concentration and was treated as the primary fatty-acid biomarker. Concentrations were reported in μmol/L. Palmitic acid C16:0, oleic acid C18:1, linoleic acid C18:2, EPA C20:5, and DHA C22:6 were evaluated as representative individual free fatty acids according to previous evidence and biological relevance.

### Principal component analysis

PCA was used to describe data-driven fatty-acid patterns. C23:0, C27:0, and C28:0 were unavailable in the first analytical batch (*n* = 99) and were excluded before PCA. The remaining 34 free fatty-acid variables had no missing values, were centered and standardized, and were entered into PCA without imputation. Derived ratios, including the free EPA/AA ratio, were not included. PC1, PC2, and PC3 were retained and interpreted using the loading matrix.

### Outcome and follow-up

Follow-up records were updated through December 31, 2025. The baseline date was the date of index hospitalization. For patients who experienced MACE, follow-up ended at the date of the first MACE event; patients without MACE were censored at the last available follow-up date. The primary outcome was MACE, defined as a composite of cardiovascular death, nonfatal myocardial infarction, clinically driven target vessel revascularization, rehospitalization for unstable angina, and ischemic stroke. Planned or staged revascularization procedures were not counted as MACE. Time-to-event was calculated from baseline to the first qualifying MACE or the last available follow-up date. Median follow-up was estimated using the reverse Kaplan–Meier method.

### Covariates and missing data

Covariates included age, sex, body mass index (BMI), smoking, hypertension, diabetes, LDL-C, HDL-C, triglycerides, eGFR, and log(Gensini score + 1). None of the variables included in the primary Cox models had missing values; therefore, no imputation was used for the primary analysis. Continuous exposures used in the Cox models were standardized as *z* scores while preserving the original variables.

### Statistical analysis

Continuous variables are presented as median [interquartile range] or mean (standard deviation), and categorical variables as numbers and percentages. Between-group comparisons used the *t* test, Wilcoxon rank-sum test, chi-square test, or Fisher exact test, as appropriate. Kaplan–Meier curves and the log-rank test were used to compare MACE-free survival according to the median free EPA/AA ratio.

Cox proportional hazards models were used to estimate HRs and 95% CIs. Model 1 adjusted for age and sex. Model 2 further adjusted for BMI, smoking, hypertension, diabetes, LDL-C, HDL-C, and triglycerides. Model 3 further adjusted for eGFR and log-transformed Gensini score and was considered the fully adjusted model. Restricted cubic splines used four knots at the 5th, 35th, 65th, and 95th percentiles. Proportional hazards assumptions were assessed using Schoenfeld residuals; when evidence of non-proportionality was present, HRs were interpreted as average associations over follow-up.

Subgroup analyses were stratified by sex, diabetes, age, and hypertension, with interaction terms used to assess effect modification. Prespecified sensitivity analyses excluded follow-up <6 months, MACE occurring <6 months, or eGFR <60 mL/min/1.73 m^2^; omitted Gensini score; and evaluated free EPA/AA quartiles. Because measurements were performed in two analytical batches using the same protocol, *post hoc* sensitivity analyses additionally adjusted for analytical batch or fitted a Cox model stratified by analytical batch. Benjamini-Hochberg FDR correction was applied to the main multivariable Cox results (24). Reporting followed the STROBE guidelines (25). All tests were two-sided, with *P* < 0.05 considered statistically significant. Analyses were performed using R version 4.4.3.

## Results

### Study population and baseline characteristics

The final analysis included 308 patients with CAD. The study flow chart is shown in Supplementary Figure S1. During a median follow-up of 48.7 months, 201 first MACE events occurred and 107 patients were censored. The distribution of first MACE components is shown in Supplementary Table S9. Among the 100 first MACE events occurring <6 months, 44 were rehospitalizations for unstable angina with target vessel revascularization, 26 were rehospitalizations for unstable angina without revascularization, 22 were cardiovascular deaths, 4 were nonfatal myocardial infarctions, 2 were ischemic strokes, 1 was unstable angina with ischemic stroke, and 1 was unstable angina with ischemic stroke and target vessel revascularization. Patients with MACE were older than those without MACE. Median free EPA concentrations were 0.29 [0.21, 0.40] and 0.28 [0.19, 0.38] μmol/L in the no-MACE and MACE groups, respectively; corresponding free AA concentrations were 4.74 [3.87, 6.11] and 4.99 [3.94, 6.27] μmol/L. The free EPA/AA ratio was numerically lower in the MACE group, although the unadjusted comparison was not statistically significant (Table 1).

**Table 1:** Baseline characteristics according to MACE status.

Variables	No MACE (*n* = 107)	MACE (*n* = 201)	*P* value
*N*	107	201	
Palmitic acid C16:0 (μmol/L)	54.48 [47.90, 67.17]	54.17 [47.22, 62.30]	0.368
Free EPA C20:5 (μmol/L)	0.29 [0.21, 0.40]	0.28 [0.19, 0.38]	0.500
Free AA C20:4 (μmol/L)	4.74 [3.87, 6.11]	4.99 [3.94, 6.27]	0.499
Free EPA/AA ratio	0.06 [0.04, 0.09]	0.05 [0.04, 0.08]	0.133
Age (years)	61.00 [53.00, 73.00]	67.00 [58.00, 78.00]	<0.001
BMI (kg/m^2^)	24.02 [21.80, 26.12]	23.29 [21.33, 25.51]	0.061
LDL-C (mmol/L)	3.06 [2.31, 3.58]	2.91 [2.21, 3.75]	0.595
HDL-C (mmol/L)	1.14 [0.92, 1.31]	1.11 [0.95, 1.32]	0.807
TG (mmol/L)	1.54 [0.94, 2.20]	1.57 [1.10, 2.13]	0.537
eGFR (mL/min/1.73 m^2^)	74.20 [56.10, 92.20]	74.10 [49.30, 91.20]	0.396
hsCRP (mg/L)	4.20 [1.96, 16.86]	4.88 [1.69, 15.03]	0.986
LVEF (%)	61.00 [53.50, 65.50]	62.00 [52.00, 66.00]	0.903
Gensini score	55.50 [32.00, 80.00]	62.00 [36.00, 93.00]	0.219
Male sex	78 (72.9%)	132 (65.7%)	0.195
Current smoking	49 (45.8%)	85 (42.3%)	0.555
Hypertension	66 (61.7%)	144 (71.6%)	0.074
Diabetes	56 (52.3%)	107 (53.2%)	0.881

Values are median [interquartile range] or *n* (%). Continuous variables were compared using the Wilcoxon rank-sum test; categorical variables were compared using the chi-square test or Fisher exact test as appropriate. All fatty-acid concentrations represent serum free (non-esterified) fatty acids. EPA, eicosapentaenoic acid; AA, arachidonic acid; BMI, body mass index; LDL-C, low-density lipoprotein cholesterol; HDL-C, high-density lipoprotein cholesterol; TG, triglycerides; eGFR, estimated glomerular filtration rate; hsCRP, high-sensitivity C-reactive protein; LVEF, left ventricular ejection fraction; MACE, major adverse cardiovascular events.

### Association between the EPA/AA ratio and MACE

In the fully adjusted model, each 1-SD increase in the free EPA/AA ratio was associated with a lower risk of MACE (HR = 0.82, 95% CI 0.70–0.96, *P* = 0.011; Table 2). The corresponding FDR-adjusted *P* value was 0.049.

**Table 2:** Multivariable Cox regression analyses for the association between serum free fatty-acid markers and MACE.

Exposure	Model 1 HR (95% CI)	Model 2 HR (95% CI)	Model 3 HR (95% CI)	Model 1 *P* value	Model 2 *P* value	Model 3 *P* value
Free EPA/AA ratio	0.82 (0.71–0.96)	0.82 (0.71–0.96)	0.82 (0.70–0.96)	0.012	0.012	0.011
Palmitic acid C16:0	0.91 (0.79–1.06)	0.92 (0.79–1.06)	0.90 (0.77–1.04)	0.218	0.238	0.161
Oleic acid C18:1	0.98 (0.86–1.12)	0.98 (0.86–1.13)	0.96 (0.84–1.11)	0.793	0.801	0.604
Linoleic acid C18:2	0.95 (0.83–1.09)	0.95 (0.83–1.09)	0.93 (0.81–1.07)	0.473	0.454	0.3
Free EPA C20:5	0.95 (0.82–1.11)	0.96 (0.82–1.12)	0.94 (0.81–1.10)	0.544	0.561	0.466
DHA C22:6	0.90 (0.78–1.05)	0.90 (0.77–1.04)	0.88 (0.76–1.03)	0.184	0.159	0.112
PC1	1.04 (0.92–1.18)	1.03 (0.91–1.18)	1.02 (0.89–1.16)	0.536	0.608	0.805
PC2	1.25 (1.09–1.43)	1.25 (1.08–1.45)	1.26 (1.08–1.45)	0.001	0.003	0.002
PC3	1.19 (1.01–1.39)	1.18 (1.01–1.39)	1.19 (1.01–1.39)	0.034	0.039	0.035

Hazard ratios are expressed per 1-SD increase in each continuous exposure. All fatty-acid concentrations represent serum free (non-esterified) fatty acids. Model 1 was adjusted for age and sex. Model 2 was additionally adjusted for BMI, current smoking, hypertension, diabetes, LDL-C, HDL-C, and triglycerides. Model 3 was additionally adjusted for eGFR and log-transformed Gensini score. PC, principal component; HR, hazard ratio; CI, confidence interval; MACE, major adverse cardiovascular events.

### Kaplan–Meier and spline analyses

MACE-free survival was better in the higher free EPA/AA group than in the lower group (log-rank *P* = 0.031; Figure 1). The restricted cubic spline for the free EPA/AA ratio showed an inverse trend, but the overall test did not reach statistical significance (*P* overall = 0.063; *P* for nonlinearity = 0.858). C16:0 showed a nonlinear association (*P* overall = 0.020; *P* for nonlinearity = 0.015; Figure 2), whereas the corresponding linear Cox association was not significant.

**Figure 1:**
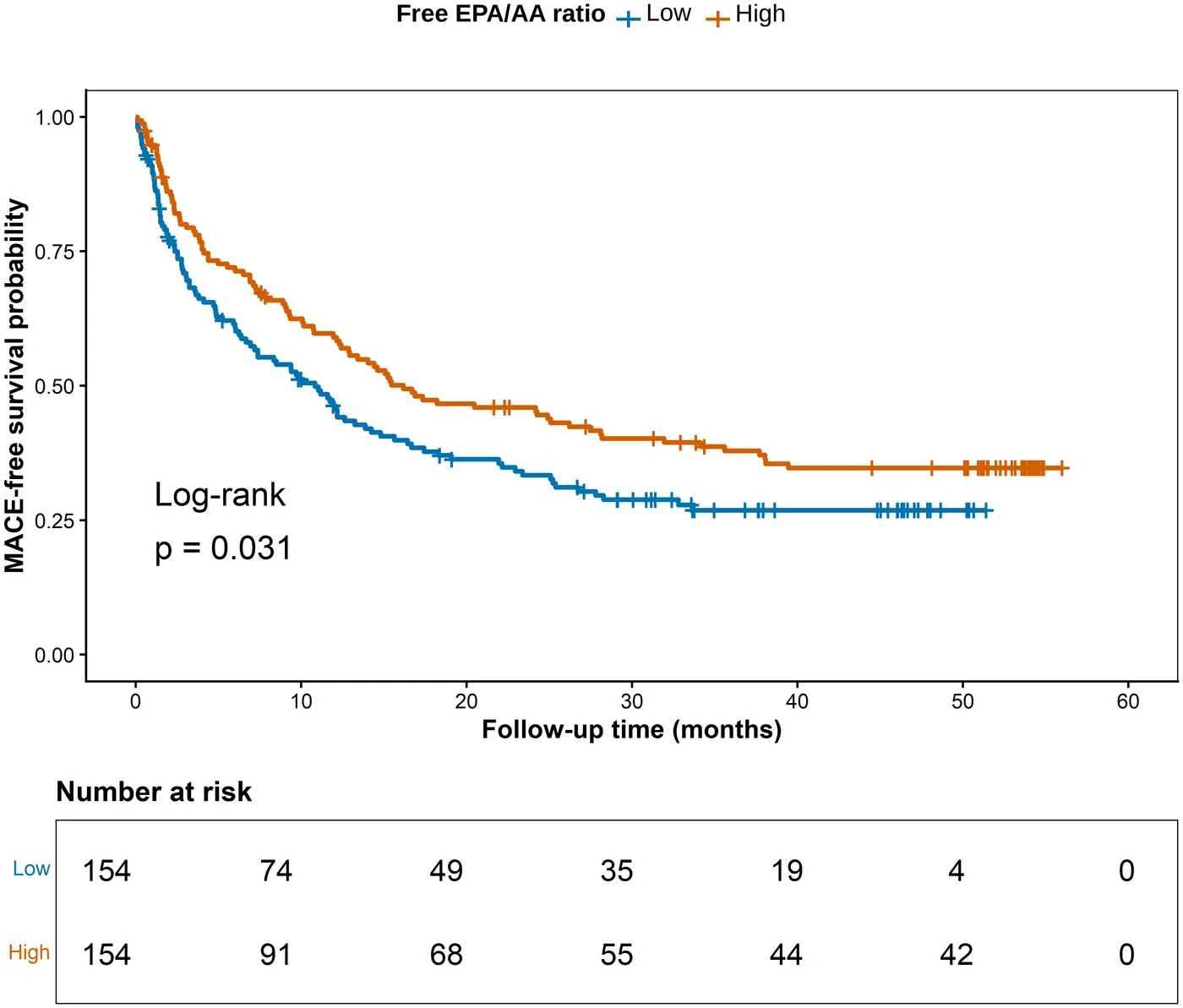
Kaplan–Meier curves for MACE-free survival according to the median free EPA/AA ratio. Patients were classified as having a low or high free EPA/AA ratio using the overall cohort median. The blue line denotes the low-ratio group and the orange line denotes the high-ratio group; tick marks indicate censored observations. The number at risk is shown below the plot. Groups were compared using the log-rank test (*P* = 0.031). EPA, eicosapentaenoic acid; AA, arachidonic acid; MACE, major adverse cardiovascular events.

**Figure 2:**
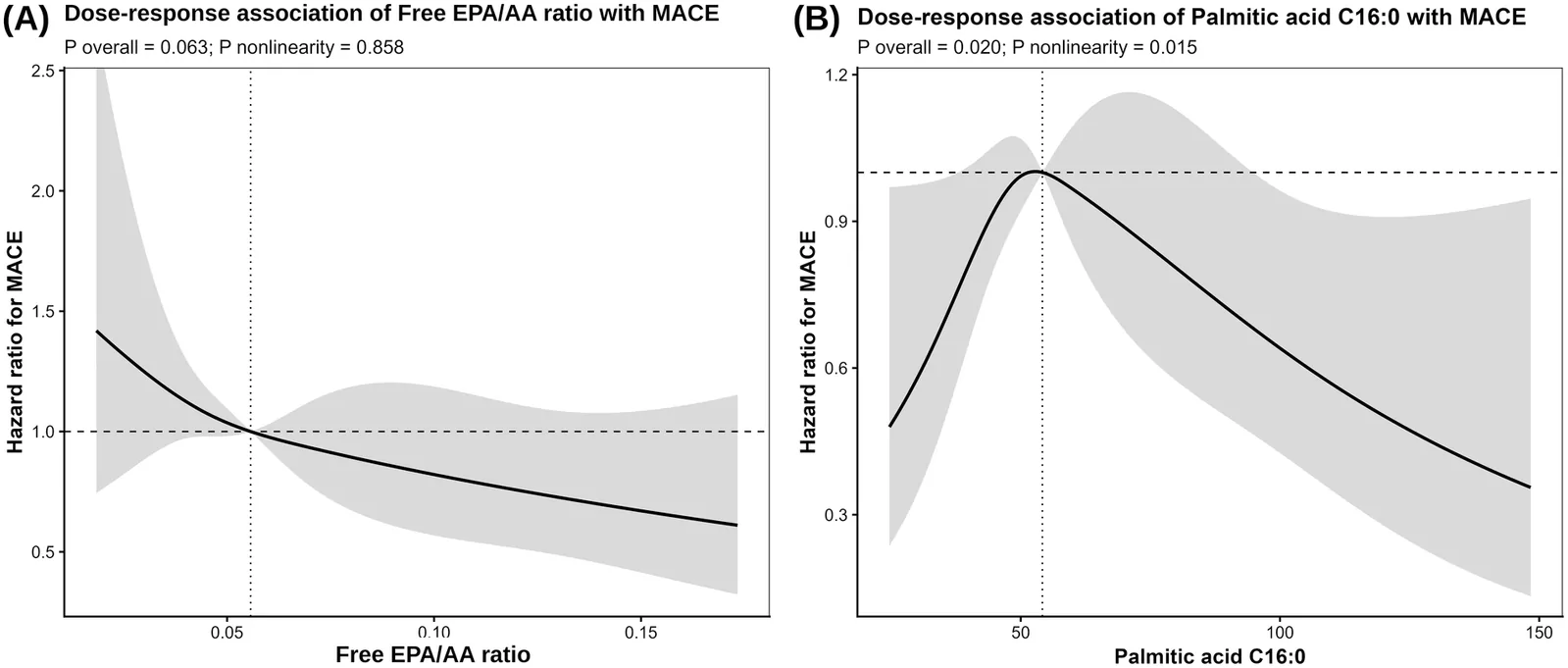
Restricted cubic spline associations of the free EPA/AA ratio and palmitic acid C16:0 with MACE. Panel **(A)** shows the free EPA/AA ratio and Panel **(B)** shows palmitic acid C16:0. Solid black lines represent adjusted hazard ratios and gray shaded areas represent 95% confidence intervals. The horizontal dashed line indicates HR = 1.0, and the vertical dotted line indicates the median exposure value used as the reference. Models were adjusted for age, sex, BMI, current smoking, hypertension, diabetes, LDL-C, HDL-C, and triglycerides. *P* values for the overall and nonlinear associations are shown in each panel.

### Subgroup and sensitivity analyses

The association differed in magnitude across age strata: the HR was 1.04 (95% CI 0.82–1.33, *P* = 0.738) in patients aged <65 years and 0.72 (95% CI 0.59–0.89, *P* = 0.002) in those aged ≥65 years, with *P* for interaction = 0.063 (Figure 3). This was treated as an exploratory finding rather than evidence of established effect modification. Prespecified exclusion analyses attenuated the primary association; after excluding MACE occurring <6 months, the HR was 0.85 (95% CI 0.70–1.03, *P* = 0.104). Additional adjustment for analytical batch yielded an HR of 0.92 (95% CI 0.77–1.10, *P* = 0.375), and stratification by analytical batch yielded an HR of 0.92 (95% CI 0.77–1.11, *P* = 0.398; Table 3).

**Figure 3:**
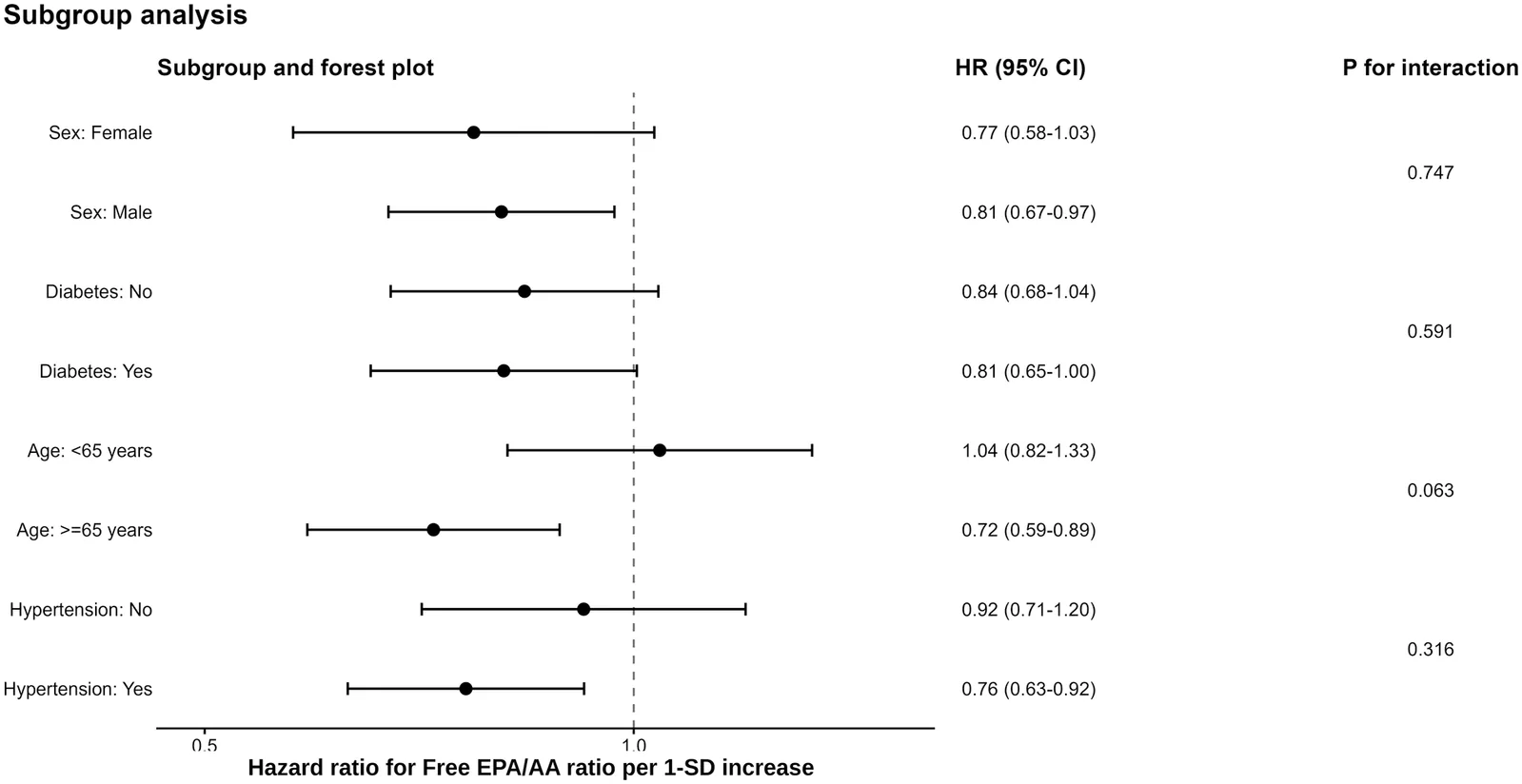
Subgroup analyses of the association between the free EPA/AA ratio and MACE. Points represent hazard ratios per 1-SD increase in the free EPA/AA ratio and horizontal lines represent 95% confidence intervals. The vertical dashed line indicates HR = 1.0. Subgroup models were adjusted for the Model 2 covariates, excluding the variable used to define the subgroup. *P* values for interaction were obtained from multiplicative interaction terms. HR, hazard ratio; CI, confidence interval; MACE, major adverse cardiovascular events.

**Table 3:** Sensitivity analyses for the association between the free EPA/AA ratio and MACE.

Sensitivity analysis	Variable	*N*	Events	HR (95% CI)	*P* value
Exclude follow-up <6 months	Free EPA/AA ratio per 1-SD	197	101	0.85 (0.70–1.03)	0.104
Exclude MACE <6 months	Free EPA/AA ratio per 1-SD	208	101	0.85 (0.70–1.03)	0.104
Exclude eGFR <60	Free EPA/AA ratio per 1-SD	204	128	0.87 (0.70–1.06)	0.172
Without Gensini	Free EPA/AA ratio per 1-SD	308	201	0.82 (0.71–0.96)	0.011
Adjusted for analytical batch	Free EPA/AA ratio per 1-SD	308	201	0.92 (0.77–1.10)	0.375
Stratified by analytical batch	Free EPA/AA ratio per 1-SD	308	201	0.92 (0.77–1.11)	0.398
Free EPA/AA quartiles	Q2 vs. Q1	308	201	0.75 (0.51–1.12)	0.162
Free EPA/AA quartiles	Q3 vs. Q1	308	201	0.67 (0.44–1.01)	0.056
Free EPA/AA quartiles	Q4 vs. Q1	308	201	0.60 (0.40–0.90)	0.014
Free EPA/AA quartiles	HR per one-quartile increment	308	201	0.85 (0.74–0.97)	0.014

Unless otherwise specified, hazard ratios are expressed per 1-SD increase in the free EPA/AA ratio and adjusted using the Model 3 covariates. The batch-adjusted model additionally included analytical batch as a covariate; the batch-stratified model allowed separate baseline hazards by analytical batch. For the quartile trend analysis, free EPA/AA quartile was modeled as an ordinal variable from 1 to 4. HR, hazard ratio; CI, confidence interval; EPA, eicosapentaenoic acid; AA, arachidonic acid; eGFR, estimated glomerular filtration rate; MACE, major adverse cardiovascular events.

### PCA-derived fatty acid patterns

PC1, PC2, and PC3 explained 41.4%, 22.9%, and 6.1% of fatty-acid variance, respectively. In the fully adjusted model, PC2 was associated with a higher risk of MACE (HR = 1.26, 95% CI 1.08–1.45, *P* = 0.002; FDR-adjusted *P* = 0.020; Table 2). PC3 showed a nominal association (HR = 1.19, 95% CI 1.01–1.39, *P* = 0.035) but was not significant after FDR correction (*P* = 0.104). PC2 was characterized by positive loadings for several long-chain free fatty acids and negative loadings for C11:0 and C12:0.

## Discussion

In this cohort of patients with angiographically confirmed CAD, a higher serum free EPA/AA ratio was associated with a lower risk of first MACE in the fully adjusted primary analysis. However, the association was attenuated after exclusion of early events and after accounting for analytical batch. The findings therefore support a possible inverse association but do not establish a robust prognostic effect.

EPA and AA are competing precursors of bioactive lipid mediators involved in vascular inflammation, platelet activation, endothelial function, and thrombosis (7, 17, 20). The ratio may reflect the balance between n-3 and n-6 pathways more directly than either concentration alone. In the present cohort, absolute free EPA and AA concentrations were similar between outcome groups, whereas the ratio showed an association after multivariable adjustment. The observational design and single baseline measurement preclude causal inference.

Observational studies have related EPA/AA measures or other omega-3 biomarkers to coronary outcomes (18, 19), whereas intervention trials of EPA or omega-3 formulations have yielded heterogeneous results (21–23). The analytical matrix is important when interpreting these data. The present study measured the serum free (non-esterified) fatty-acid fraction without hydrolysis, whereas many published EPA/AA values are derived from total serum or plasma fatty acids. Absolute values and thresholds from these approaches are therefore not interchangeable. These studies are considered here as biological and clinical context rather than as direct numerical comparators.

The restricted cubic spline analysis showed an inverse trend for the free EPA/AA ratio, although the overall test did not reach statistical significance. The association was also attenuated after exclusion of MACE occurring <6 months, indicating sensitivity to event timing. Among the early events, rehospitalization for unstable angina, with or without clinically driven target vessel revascularization, was common. Planned or staged revascularization was not counted as MACE, but the broad composite endpoint should still be considered when interpreting the result.

The age subgroup analysis also warrants a cautious interpretation. The inverse association was apparent in patients aged ≥65 years but not in those aged <65 years; however, the interaction *P* value was 0.063. This observation should be regarded as exploratory and requires confirmation. PCA provided additional information on the overall free fatty-acid profile. PC2 was associated with a higher risk of MACE, but PCA components are data-driven and sample-dependent and should be viewed as descriptive metabolic patterns rather than established biomarkers.

The main strengths of this study include angiographic confirmation of CAD, long-term follow-up, and measurement of individual serum free fatty acids by LC-MS/MS. Several limitations should be considered. First, this was a single-center observational study, and inclusion depended on written informed consent and availability of a suitable stored fasting serum sample; residual confounding and selection bias cannot be excluded. Second, free fatty acids were measured only at baseline. Although sampling was performed after fasting and before angiography and heparin administration, detailed information on diet, medication changes, and lifestyle factors was not fully available. Third, detailed historical analyte-specific analytical validation data, including intra- and inter-assay coefficients of variation, recovery, accuracy, limits of detection/quantification, and predefined QC acceptance criteria, were unavailable. The same LC-MS/MS protocol was used in two analytical batches, but shared cross-batch QC or replicate reference samples were not available. Because the free EPA/AA distribution differed between batches and batch-adjusted analyses attenuated the association, patient heterogeneity and potential inter-batch analytical variation cannot be fully separated. Fourth, MACE was a broad composite endpoint, and early unstable-angina events and clinically driven revascularization contributed substantially to the event count. Fifth, the PCA findings were exploratory, and some proportional hazards tests suggested time-varying effects. Independent validation is required.

## Conclusions

In the primary multivariable analysis, a higher serum free EPA/AA ratio was associated with a lower long-term risk of MACE in patients with angiographically confirmed CAD. The association was attenuated in analyses accounting for early events and analytical batch. These findings should be considered hypothesis-generating and require confirmation in independent cohorts.

## Data Availability

The datasets presented in this article are not readily available because of institutional data-governance requirements and patient privacy considerations. Deidentified data may be made available upon reasonable request, subject to institutional approval and applicable ethical requirements. Requests to access the datasets should be directed to fsdrwu@126.com.
