# Supplementary material for: Serum free eicosapentaenoic acid-to-arachidonic acid ratio and long-term risk of major adverse cardiovascular events in patients with coronary artery disease
Source: Front Cardiovasc Med. 2026 Sep 29;13:1944383. doi: 10.3389/fcvm.2026.1944383 (PMC13658503; doi:10.3389/fcvm.2026.1944383)
Supplement: Supplementary file 1 [file Datasheet1.docx]

Supplementary Tables

**Supplementary Table S1. Baseline characteristics according to quartiles of the free EPA/AA ratio**

| **Variables** | **Q1 (n=77)** | **Q2 (n=77)** | **Q3 (n=77)** | **Q4 (n=77)** | **P value** |
| --- | --- | --- | --- | --- | --- |
| N | 77 | 77 | 77 | 77 |  |
| Palmitic acid C16:0 (μmol/L) | 50.41 [42.61, 56.75] | 52.77 [47.74, 59.39] | 55.54 [48.70, 67.32] | 61.36 [49.26, 78.49] | <0.001 |
| Free EPA C20:5 (μmol/L) | 0.16 [0.12, 0.19] | 0.24 [0.19, 0.30] | 0.36 [0.29, 0.42] | 0.42 [0.32, 0.67] | <0.001 |
| Free AA C20:4 (μmol/L) | 5.14 [4.17, 6.38] | 4.99 [4.20, 6.17] | 5.22 [4.21, 6.38] | 3.94 [2.82, 5.36] | <0.001 |
| Free EPA/AA ratio | 0.03 [0.03, 0.04] | 0.05 [0.04, 0.05] | 0.07 [0.06, 0.07] | 0.11 [0.10, 0.13] | <0.001 |
| Age (years) | 66.00 [55.00, 78.00] | 66.00 [57.00, 77.00] | 63.00 [56.00, 75.00] | 66.00 [58.00, 75.00] | 0.836 |
| BMI (kg/m²) | 22.40 [21.11, 24.68] | 24.06 [21.64, 25.86] | 24.03 [21.89, 26.17] | 23.53 [21.80, 25.51] | 0.199 |
| LDL-C (mmol/L) | 2.98 [2.31, 3.59] | 3.12 [2.39, 3.84] | 2.82 [2.32, 3.97] | 2.75 [2.06, 3.43] | 0.340 |
| HDL-C (mmol/L) | 1.18 [0.98, 1.32] | 1.14 [0.98, 1.35] | 1.13 [0.92, 1.33] | 1.06 [0.90, 1.23] | 0.314 |
| TG (mmol/L) | 1.40 [1.05, 1.94] | 1.57 [1.01, 2.32] | 1.56 [1.15, 2.23] | 1.58 [1.00, 2.19] | 0.600 |
| eGFR (mL/min/1.73 m²) | 79.70 [58.40, 97.80] | 73.10 [40.70, 86.80] | 69.80 [48.40, 90.10] | 73.80 [56.90, 88.40] | 0.070 |
| hsCRP (mg/L) | 6.08 [2.75, 39.55] | 4.90 [1.96, 22.10] | 3.40 [1.34, 7.40] | 4.30 [1.87, 10.04] | 0.030 |
| LVEF (%) | 61.00 [52.00, 65.00] | 60.00 [50.00, 65.00] | 63.00 [52.00, 66.00] | 63.00 [57.00, 67.00] | 0.074 |
| Gensini score | 55.50 [37.00, 88.00] | 56.50 [38.00, 84.00] | 69.00 [32.00, 94.00] | 54.00 [33.00, 84.00] | 0.635 |
| Male sex | 58 (75.3%) | 53 (68.8%) | 48 (62.3%) | 51 (66.2%) | 0.366 |
| Current smoking | 46 (59.7%) | 38 (49.4%) | 28 (36.4%) | 22 (28.6%) | <0.001 |
| Hypertension | 50 (64.9%) | 56 (72.7%) | 52 (67.5%) | 52 (67.5%) | 0.768 |
| Diabetes | 49 (63.6%) | 43 (55.8%) | 39 (50.6%) | 32 (41.6%) | 0.047 |

Values are median [interquartile range] or n (%). P values were obtained using the Kruskal–Wallis test for continuous variables and the χ² test or Fisher exact test for categorical variables as appropriate. All fatty-acid concentrations represent serum free (non-esterified) fatty acids.

**Supplementary Table S2. Restricted cubic spline tests**

| **Exposure** | **Knot locations** | **P overall** | **P for nonlinearity** |
| --- | --- | --- | --- |
| Free EPA/AA ratio | 0.0258, 0.0456, 0.0696, 0.139 | 0.063 | 0.858 |
| Palmitic acid C16:0 | 32.3227, 49.6514, 59.1368, 110.0618 | 0.020 | 0.015 |
| Arachidonic acid C20:4 | 2.637, 4.3082, 5.5929, 8.697 | 0.204 | 0.531 |
| EPA C20:5 | 0.1124, 0.2272, 0.343, 0.7718 | 0.672 | 0.550 |
| DHA C22:6 | 0.5567, 1.0096, 1.4523, 3.6276 | 0.051 | 0.062 |

Restricted cubic spline models used four knots at the 5th, 35th, 65th, and 95th percentiles of each exposure and were adjusted for age, sex, BMI, current smoking, hypertension, diabetes, LDL-C, HDL-C, and triglycerides. P overall tests the overall association; P for nonlinearity compares the spline model with the corresponding linear model. All fatty-acid concentrations represent serum free (non-esterified) fatty acids.

**Supplementary Table S3. Subgroup analyses of the association between the free EPA/AA ratio and MACE**

| **Subgroup** | **Category** | **N** | **Events** | **HR (95% CI)** | **P value** | **P for interaction** |
| --- | --- | --- | --- | --- | --- | --- |
| Sex | Female | 98 | 69 | 0.77 (0.58–1.03) | 0.082 | 0.747 |
| Sex | Male | 210 | 132 | 0.81 (0.67–0.97) | 0.022 |  |
| Diabetes | No | 145 | 94 | 0.84 (0.68–1.04) | 0.110 | 0.591 |
| Diabetes | Yes | 163 | 107 | 0.81 (0.65–1.00) | 0.056 |  |
| Age | <65 years | 147 | 86 | 1.04 (0.82–1.33) | 0.738 | 0.063 |
| Age | >=65 years | 161 | 115 | 0.72 (0.59–0.89) | 0.002 |  |
| Hypertension | No | 98 | 57 | 0.92 (0.71–1.20) | 0.545 | 0.316 |
| Hypertension | Yes | 210 | 144 | 0.76 (0.63–0.92) | 0.005 |  |

Hazard ratios are expressed per 1-SD increase in the free EPA/AA ratio. Subgroup models were adjusted for the Model 2 covariates, excluding the variable used to define the subgroup. P for interaction was obtained from the corresponding multiplicative interaction term. HR, hazard ratio; CI, confidence interval.

**Supplementary Table S4. Missingness of variables included in the primary multivariable Cox model**

| **Variable** | **Missing, n** | **Missing, %** |
| --- | --- | --- |
| Follow-up time | 0 | 0.0 |
| MACE status | 0 | 0.0 |
| Free EPA/AA ratio | 0 | 0.0 |
| Age | 0 | 0.0 |
| Sex | 0 | 0.0 |
| BMI | 0 | 0.0 |
| Current smoking | 0 | 0.0 |
| Hypertension | 0 | 0.0 |
| Diabetes | 0 | 0.0 |
| LDL-C | 0 | 0.0 |
| HDL-C | 0 | 0.0 |
| Triglycerides | 0 | 0.0 |
| eGFR | 0 | 0.0 |
| log(Gensini score + 1) | 0 | 0.0 |

No variable included in the primary multivariable Cox model had missing values; therefore, no imputation was used for the primary analysis.

**Supplementary Table S5. Missingness of measured free fatty acids**

| **Fatty acid** | **Missing, n** | **Missing, %** |
| --- | --- | --- |
| C23:0 | 99 | 32.1 |
| C27:0 | 99 | 32.1 |
| C28:0 | 99 | 32.1 |
| C11:0 | 0 | 0.0 |
| C12:0 | 0 | 0.0 |
| C14:0 | 0 | 0.0 |
| C14:1 | 0 | 0.0 |
| C15:0 | 0 | 0.0 |
| C16:0 | 0 | 0.0 |
| C16:1 | 0 | 0.0 |
| C17:0 | 0 | 0.0 |
| C17:1 | 0 | 0.0 |
| C18:0 | 0 | 0.0 |
| C18:1 | 0 | 0.0 |
| C18:2 | 0 | 0.0 |
| C18:3 | 0 | 0.0 |
| C18:4 | 0 | 0.0 |
| C20:0 | 0 | 0.0 |
| C20:1 | 0 | 0.0 |
| C20:2 | 0 | 0.0 |
| C20:3 | 0 | 0.0 |
| C20:4 | 0 | 0.0 |
| C20:5 | 0 | 0.0 |
| C21:0 | 0 | 0.0 |
| C22:0 | 0 | 0.0 |
| C22:1 | 0 | 0.0 |
| C22:2 | 0 | 0.0 |
| C22:3 | 0 | 0.0 |
| C22:4 | 0 | 0.0 |
| C22:5 | 0 | 0.0 |
| C22:6 | 0 | 0.0 |
| C24:0 | 0 | 0.0 |
| C24:1 | 0 | 0.0 |
| C25:0 | 0 | 0.0 |
| C25:1 | 0 | 0.0 |
| C26:0 | 0 | 0.0 |
| C26:1 | 0 | 0.0 |

C23:0, C27:0, and C28:0 were unavailable in the first analytical batch (n=99) and were excluded from the principal component analysis. The remaining fatty acids used in the principal component analysis were complete.

**Supplementary Table S6A. Variance explained by principal components**

| **Component** | **Eigenvalue** | **Variance explained, %** | **Cumulative variance, %** |
| --- | --- | --- | --- |
| PC1 | 14.060 | 41.35 | 41.35 |
| PC2 | 7.780 | 22.88 | 64.24 |
| PC3 | 2.082 | 6.12 | 70.36 |
| PC4 | 1.819 | 5.35 | 75.71 |
| PC5 | 1.514 | 4.45 | 80.16 |
| PC6 | 1.036 | 3.05 | 83.21 |
| PC7 | 0.953 | 2.80 | 86.01 |
| PC8 | 0.701 | 2.06 | 88.07 |
| PC9 | 0.624 | 1.83 | 89.91 |
| PC10 | 0.509 | 1.50 | 91.40 |
| PC11 | 0.457 | 1.34 | 92.75 |
| PC12 | 0.373 | 1.10 | 93.84 |
| PC13 | 0.329 | 0.97 | 94.81 |
| PC14 | 0.247 | 0.73 | 95.54 |
| PC15 | 0.226 | 0.66 | 96.20 |
| PC16 | 0.196 | 0.58 | 96.78 |
| PC17 | 0.163 | 0.48 | 97.26 |
| PC18 | 0.148 | 0.44 | 97.69 |
| PC19 | 0.116 | 0.34 | 98.03 |
| PC20 | 0.104 | 0.30 | 98.34 |
| PC21 | 0.088 | 0.26 | 98.60 |
| PC22 | 0.074 | 0.22 | 98.81 |
| PC23 | 0.068 | 0.20 | 99.01 |
| PC24 | 0.061 | 0.18 | 99.19 |
| PC25 | 0.050 | 0.15 | 99.34 |
| PC26 | 0.042 | 0.12 | 99.46 |
| PC27 | 0.039 | 0.12 | 99.58 |
| PC28 | 0.034 | 0.10 | 99.68 |
| PC29 | 0.029 | 0.09 | 99.76 |
| PC30 | 0.026 | 0.08 | 99.84 |
| PC31 | 0.018 | 0.05 | 99.89 |
| PC32 | 0.014 | 0.04 | 99.93 |
| PC33 | 0.013 | 0.04 | 99.97 |
| PC34 | 0.011 | 0.03 | 100.00 |

**Supplementary Table S6B. Principal component loading matrix**

| **Fatty acid** | **PC1** | **PC2** | **PC3** |
| --- | --- | --- | --- |
| C11:0 | 0.040 | -0.315 | -0.198 |
| C12:0 | 0.143 | -0.210 | -0.165 |
| C14:0 | 0.236 | -0.084 | -0.026 |
| C14:1 | 0.208 | -0.140 | -0.027 |
| C15:0 | 0.159 | 0.052 | 0.159 |
| C16:0 | 0.212 | -0.160 | -0.187 |
| C16:1 | 0.237 | -0.094 | 0.023 |
| C17:0 | 0.179 | 0.200 | 0.178 |
| C17:1 | 0.251 | 0.000 | 0.124 |
| C18:0 | 0.177 | 0.055 | -0.181 |
| C18:1 | 0.240 | -0.101 | -0.145 |
| C18:2 | 0.239 | -0.091 | -0.147 |
| C18:3 | 0.220 | 0.001 | -0.124 |
| C18:4 | 0.188 | -0.028 | 0.082 |
| C20:0 | 0.093 | 0.111 | -0.444 |
| C20:1 | 0.204 | 0.154 | -0.009 |
| C20:2 | 0.217 | 0.152 | 0.130 |
| C20:3 | 0.214 | 0.097 | 0.134 |
| C20:4 | 0.186 | 0.074 | 0.005 |
| C20:5 | 0.156 | -0.155 | 0.068 |
| C21:0 | -0.007 | 0.284 | 0.167 |
| C22:0 | 0.019 | 0.231 | -0.278 |
| C22:1 | 0.090 | 0.138 | -0.194 |
| C22:2 | 0.189 | 0.186 | 0.084 |
| C22:3 | 0.142 | 0.261 | 0.210 |
| C22:4 | 0.232 | 0.065 | 0.090 |
| C22:5 | 0.228 | -0.093 | 0.074 |
| C22:6 | 0.211 | -0.131 | 0.054 |
| C24:0 | 0.007 | 0.299 | -0.165 |
| C24:1 | 0.087 | 0.262 | -0.006 |
| C25:0 | 0.005 | 0.105 | -0.387 |
| C25:1 | -0.060 | 0.199 | -0.125 |
| C26:0 | -0.017 | 0.320 | -0.030 |
| C26:1 | -0.005 | 0.188 | -0.294 |

**Supplementary Table S6C. Ten largest absolute loadings for PC1–PC3**

| **Fatty acid** | **Component** | **Loading** | **Absolute loading** |
| --- | --- | --- | --- |
| C17:1 | PC1 | 0.251 | 0.251 |
| C18:1 | PC1 | 0.240 | 0.240 |
| C18:2 | PC1 | 0.239 | 0.239 |
| C16:1 | PC1 | 0.237 | 0.237 |
| C14:0 | PC1 | 0.236 | 0.236 |
| C22:4 | PC1 | 0.232 | 0.232 |
| C22:5 | PC1 | 0.228 | 0.228 |
| C18:3 | PC1 | 0.220 | 0.220 |
| C20:2 | PC1 | 0.217 | 0.217 |
| C20:3 | PC1 | 0.214 | 0.214 |
| C26:0 | PC2 | 0.320 | 0.320 |
| C11:0 | PC2 | -0.315 | 0.315 |
| C24:0 | PC2 | 0.299 | 0.299 |
| C21:0 | PC2 | 0.284 | 0.284 |
| C24:1 | PC2 | 0.262 | 0.262 |
| C22:3 | PC2 | 0.261 | 0.261 |
| C22:0 | PC2 | 0.231 | 0.231 |
| C12:0 | PC2 | -0.210 | 0.210 |
| C17:0 | PC2 | 0.200 | 0.200 |
| C25:1 | PC2 | 0.199 | 0.199 |
| C20:0 | PC3 | -0.444 | 0.444 |
| C25:0 | PC3 | -0.387 | 0.387 |
| C26:1 | PC3 | -0.294 | 0.294 |
| C22:0 | PC3 | -0.278 | 0.278 |
| C22:3 | PC3 | 0.210 | 0.210 |
| C11:0 | PC3 | -0.198 | 0.198 |
| C22:1 | PC3 | -0.194 | 0.194 |
| C16:0 | PC3 | -0.187 | 0.187 |
| C18:0 | PC3 | -0.181 | 0.181 |
| C17:0 | PC3 | 0.178 | 0.178 |

**Supplementary Table S6D. Exploratory individual Cox analyses of the ten fatty acids with the largest absolute PC2 loadings**

| **Fatty acid** | **PC2 loading** | **Direction** | **N** | **Events** | **HR (95% CI)** | **P value** | **FDR-adjusted P value** |
| --- | --- | --- | --- | --- | --- | --- | --- |
| C26:0 | 0.320 | Positive | 308 | 201 | 1.17 (1.02–1.35) | 0.025 | 0.041 |
| C11:0 | -0.315 | Negative | 308 | 201 | 0.74 (0.63–0.87) | <0.001 | 0.003 |
| C24:0 | 0.299 | Positive | 308 | 201 | 1.07 (0.94–1.22) | 0.319 | 0.354 |
| C21:0 | 0.284 | Positive | 308 | 201 | 1.17 (1.01–1.35) | 0.037 | 0.046 |
| C24:1 | 0.262 | Positive | 308 | 201 | 1.15 (1.01–1.31) | 0.034 | 0.046 |
| C22:3 | 0.261 | Positive | 308 | 201 | 1.26 (1.09–1.45) | 0.001 | 0.007 |
| C22:0 | 0.231 | Positive | 308 | 201 | 0.99 (0.87–1.13) | 0.876 | 0.876 |
| C12:0 | -0.210 | Negative | 308 | 201 | 0.74 (0.61–0.90) | 0.003 | 0.01 |
| C17:0 | 0.200 | Positive | 308 | 201 | 1.21 (1.05–1.41) | 0.009 | 0.023 |
| C25:1 | 0.199 | Positive | 308 | 201 | 1.19 (1.04–1.36) | 0.012 | 0.025 |

Hazard ratios are expressed per 1-SD increase in each fatty acid and adjusted using the Model 3 covariates. FDR-adjusted P values were calculated using the Benjamini–Hochberg method across the ten exploratory analyses.

**Supplementary Table S7. Schoenfeld residual tests of the proportional hazards assumption**

| **Exposure** | **Test** | **χ²** | **P value** |
| --- | --- | --- | --- |
| Free EPA/AA ratio | Exposure-specific | 3.558 | 0.059 |
| Free EPA/AA ratio | Global model | 29.261 | 0.004 |
| Palmitic acid C16:0 | Exposure-specific | 0.217 | 0.641 |
| Palmitic acid C16:0 | Global model | 26.384 | 0.009 |
| Oleic acid C18:1 | Exposure-specific | 2.649 | 0.104 |
| Oleic acid C18:1 | Global model | 28.283 | 0.005 |
| Linoleic acid C18:2 | Exposure-specific | 2.347 | 0.126 |
| Linoleic acid C18:2 | Global model | 27.698 | 0.006 |
| EPA C20:5 | Exposure-specific | 0.016 | 0.898 |
| EPA C20:5 | Global model | 26.699 | 0.009 |
| DHA C22:6 | Exposure-specific | 2.579 | 0.108 |
| DHA C22:6 | Global model | 28.657 | 0.004 |
| PC1 | Exposure-specific | 7.692 | 0.006 |
| PC1 | Global model | 31.730 | 0.002 |
| PC2 | Exposure-specific | 11.547 | 0.001 |
| PC2 | Global model | 33.434 | 0.001 |
| PC3 | Exposure-specific | 0.005 | 0.943 |
| PC3 | Global model | 25.860 | 0.011 |

Exposure-specific and global proportional-hazards tests were obtained using scaled Schoenfeld residuals from each Model 3 Cox regression.

**Supplementary Table S8. Benjamini–Hochberg FDR-adjusted P values for the multivariable Cox analyses**

| **Exposure** | **Model** | **N** | **Events** | **HR (95% CI)** | **Raw P value** | **FDR-adjusted P value** |
| --- | --- | --- | --- | --- | --- | --- |
| Free EPA/AA ratio | Model 1 | 308 | 201 | 0.82 (0.71–0.96) | 0.012 | 0.052 |
| Free EPA/AA ratio | Model 2 | 308 | 201 | 0.82 (0.71–0.96) | 0.012 | 0.053 |
| Free EPA/AA ratio | Model 3 | 308 | 201 | 0.82 (0.70–0.96) | 0.011 | 0.049 |
| Palmitic acid C16:0 | Model 1 | 308 | 201 | 0.91 (0.79–1.06) | 0.218 | 0.393 |
| Palmitic acid C16:0 | Model 2 | 308 | 201 | 0.92 (0.79–1.06) | 0.238 | 0.429 |
| Palmitic acid C16:0 | Model 3 | 308 | 201 | 0.90 (0.77–1.04) | 0.161 | 0.290 |
| Oleic acid C18:1 | Model 1 | 308 | 201 | 0.98 (0.86–1.12) | 0.793 | 0.793 |
| Oleic acid C18:1 | Model 2 | 308 | 201 | 0.98 (0.86–1.13) | 0.801 | 0.801 |
| Oleic acid C18:1 | Model 3 | 308 | 201 | 0.96 (0.84–1.11) | 0.604 | 0.679 |
| Linoleic acid C18:2 | Model 1 | 308 | 201 | 0.95 (0.83–1.09) | 0.473 | 0.612 |
| Linoleic acid C18:2 | Model 2 | 308 | 201 | 0.95 (0.83–1.09) | 0.454 | 0.680 |
| Linoleic acid C18:2 | Model 3 | 308 | 201 | 0.93 (0.81–1.07) | 0.300 | 0.450 |
| EPA C20:5 | Model 1 | 308 | 201 | 0.95 (0.82–1.11) | 0.544 | 0.612 |
| EPA C20:5 | Model 2 | 308 | 201 | 0.96 (0.82–1.12) | 0.561 | 0.684 |
| EPA C20:5 | Model 3 | 308 | 201 | 0.94 (0.81–1.10) | 0.466 | 0.600 |
| DHA C22:6 | Model 1 | 308 | 201 | 0.90 (0.78–1.05) | 0.184 | 0.393 |
| DHA C22:6 | Model 2 | 308 | 201 | 0.90 (0.77–1.04) | 0.159 | 0.358 |
| DHA C22:6 | Model 3 | 308 | 201 | 0.88 (0.76–1.03) | 0.112 | 0.252 |
| PC1 | Model 1 | 308 | 201 | 1.04 (0.92–1.18) | 0.536 | 0.612 |
| PC1 | Model 2 | 308 | 201 | 1.03 (0.91–1.18) | 0.608 | 0.684 |
| PC1 | Model 3 | 308 | 201 | 1.02 (0.89–1.16) | 0.805 | 0.805 |
| PC2 | Model 1 | 308 | 201 | 1.25 (1.09–1.43) | 0.001 | 0.013 |
| PC2 | Model 2 | 308 | 201 | 1.25 (1.08–1.45) | 0.003 | 0.025 |
| PC2 | Model 3 | 308 | 201 | 1.26 (1.08–1.45) | 0.002 | 0.020 |
| PC3 | Model 1 | 308 | 201 | 1.19 (1.01–1.39) | 0.034 | 0.102 |
| PC3 | Model 2 | 308 | 201 | 1.18 (1.01–1.39) | 0.039 | 0.117 |
| PC3 | Model 3 | 308 | 201 | 1.19 (1.01–1.39) | 0.035 | 0.104 |

FDR-adjusted P values were calculated separately within each adjustment model across the fatty-acid markers and principal components shown in Table 2.

**Supplementary Table S9. Distribution of first MACE components**

| **First MACE component pattern** | **n (%)** |
| --- | --- |
| Rehospitalization for unstable angina | 89 (44.3) |
| Rehospitalization for unstable angina + Target vessel revascularization | 62 (30.8) |
| Cardiovascular death | 36 (17.9) |
| Nonfatal myocardial infarction | 9 (4.5) |
| Ischemic stroke | 3 (1.5) |
| Rehospitalization for unstable angina + Ischemic stroke | 1 (0.5) |
| Rehospitalization for unstable angina + Ischemic stroke + Target vessel revascularization | 1 (0.5) |
| Total first MACE events | 201 (100.0) |

Only the first qualifying MACE was used for time-to-event analyses. Planned or staged revascularization procedures were not counted as MACE; target-vessel revascularization was recorded only when clinically driven in the context of a qualifying subsequent ischemic event.

# Supplementary Figure Legends

**Supplementary Figure S1. Study flow chart.**

The study database contained 311 screened patients. Three patients without angiographically confirmed coronary artery disease were excluded, leaving 308 patients in the final analysis cohort; 201 experienced a first MACE and 107 were censored during follow-up. CAD, coronary artery disease; MACE, major adverse cardiovascular events.

**Supplementary Figure S2. Univariable Cox analyses of fatty-acid markers and principal components.**

Points represent hazard ratios per 1-SD increase in each exposure and horizontal lines represent 95% confidence intervals. The vertical dashed line indicates HR=1.0. PC, principal component; HR, hazard ratio; CI, confidence interval. All individual fatty-acid concentrations represent serum free (non-esterified) fatty acids.

**Supplementary Figure S3. Restricted cubic spline analyses for individual fatty-acid exposures.**

Panels show (A) the free EPA/AA ratio, (B) palmitic acid C16:0, (C) arachidonic acid C20:4, (D) EPA C20:5, and (E) DHA C22:6. Solid black lines represent adjusted hazard ratios and gray shaded areas represent 95% confidence intervals. Horizontal dashed lines indicate HR=1.0 and vertical dotted lines indicate median exposure values used as references. Models were adjusted for age, sex, BMI, current smoking, hypertension, diabetes, LDL-C, HDL-C, and triglycerides. P values for the overall and nonlinear associations are shown in each panel. All individual fatty-acid concentrations represent serum free (non-esterified) fatty acids.
